# Editorial: Exploring the impact of inflammatory processes on the course of acute myocardial infarction: pathways and therapies

**DOI:** 10.3389/fcvm.2026.2001946

**Published:** 2026-10-06

**Authors:** Manan Pareek, Tarek Bekfani, Mihaela Popescu

**Affiliations:** 1 Department of Cardiology, Rigshospitalet, University of Copenhagen, Copenhagen, Denmark; 2 Faculty of Medicine, University Hospital Magdeburg, Magdeburg, Germany; 3 Elias Cardiology Department, Carol Davila University of Medicine and Pharmacy, Bucharest, Romania

**Keywords:** acute myocardial infarction, biomarkers, cardiac repair, cardioprotection, inflammation, ischemia-reperfusion injury

Acute myocardial infarction (AMI) triggers an inflammatory cascade that is necessary to clear necrotic debris and initiate myocardial repair, yet may prove deleterious when it becomes excessive, extending injury and delaying healing. Within hours of coronary occlusion, innate immune cells are recruited to orchestrate this process, and an excessive or poorly timed response predisposes patients to adverse remodelling. This double, time-dependent character of inflammation motivated this Research Topic: to bring together mechanistic, translational, and clinical perspectives which might help define when inflammation helps, when it harms, and how it might be safely modulated.

The nine contributions assembled here span molecular signalling, natural and traditional compounds, drug-delivery innovation, and clinical biomarkers. Together, they provide a more coherent picture of the inflammatory trajectory of AMI than any single study might offer.

Several papers have examined the mechanistic pathway that couples ischemic injury to inflammatory amplification and repair. Ma and Jiang have provided a comprehensive review of NF-κB signalling, underlining its central role in cytokine production, leukocyte recruitment, and cardiomyocyte apoptosis after infarction. They also outline the pharmacological strategies to restrain its more deleterious effects without silencing the reparative activity (Ma and Jiang). In addition, Zhang et al. reviewed the contribution of the Notch pathway in cardiac repair, treating it as a promising but still underexploited target for promoting angiogenesis and limiting fibrosis after acute infarction (Zhang et al.). Li et al. reported that upregulation of Hoxb4 expression by a traditional formula (Xuan Bi Tong Yu Fang) confers cardioprotection in ischaemia-reperfusion injury through repression of Wnt/β-catenin signalling. This affords a mechanistic anchor for a compound that has been long used empirically (Li et al.).

Traditional and plant-derived therapeutics featured in this collection demonstrate a broader interest in multitarget strategies that single-pathway drugs have struggled to match. Sun et al. contributed a wide-ranging review and proposed matching traditional Chinese medicine compounds to the spatiotemporal and endotype-specific features of ischaemia-reperfusion injury, an appealing framework for reconciling heterogeneous trial results with heterogeneous patient biology (Sun et al.). Li et al. reported that Helianthus annuus L. extract attenuates myocardial ischemia by suppressing inflammation and oxidative stress. This supplies experimental support to the growing body of phytotherapeutic evidence. However, clinical translation remains an open question (Li et al.).

A second cluster of articles addresses the practical challenge that motivated this Research Topic: how, and when, to measure inflammation in a clinically useful way. Melekoglu et al. reported that serum endocan, a marker of endothelial activation, is elevated in patients with non-ST-elevation myocardial infarction and may aid in risk stratification beyond common markers (Melekoglu et al.). Li et al. extend this line of inquiry to the intensive care setting, demonstrating that inflammatory marker profiles track long-term prognosis in critically ill NSTEMI patients, strengthening the concept that a single admission value is less informative than a trajectory (Li et al.). Gárate et al. contributed a longitudinal observational study of alpha-CGRP, a neuropeptide with vasodilatory and anti-inflammatory properties, which revealed dynamic changes during AMI, suggesting a compensatory or protective response that is worthy of further characterization (Gárate et al.). Together, these studies reinforce a recurring message of this Research Topic: timing and trajectory, not a single cut-off, are what gives inflammatory biomarkers prognostic value.

Finally, Li et al. reviewed nanoparticle-based drug delivery systems designed to target inflammatory and immune mechanisms in AMI and investigated strategies to concentrate anti-inflammatory or reparative payloads at the infarct site while limiting systemic exposure (Li et al.). This work, together with the mechanistic and natural-compound studies above, points toward a shared translational challenge for the field: moving from pathway identification and preclinical proof-of-concept toward interventions with defined therapeutic windows, validated targets, and dosing regimens that curb harmful inflammation without impairing the repair process it also sustains.

Taken as a whole, this Research Topic reaffirms that inflammation in AMI cannot be treated as a single, uniformly harmful process to be blanketly suppressed. It is compartmentalized in time, cell type, and molecular pathway, and effective intervention will likely depend on choosing the right target, the right compound, and the right window for the right patient. Several gaps remain clear from these contributions: the biomarker studies here, promising as they are, come from single-center or modest cohorts and require multicenter validation before they can inform practice; the mechanistic and natural-compound findings are largely preclinical and await rigorous clinical testing; and nanoparticle delivery systems, while conceptually elegant, still face major challenges in manufacturing, safety, and regulatory translation. We hope that this collection operates as both a synthesis of the current understanding and a roadmap for the studies still needed.

We thank all the authors for their valuable contributions and the reviewers for their constructive evaluations.

